# Prevalence of and Factors Associated with Lens Opacities in a Korean Adult Population with and without Diabetes: The 2008–2009 Korea National Health and Nutrition Examination Survey

**DOI:** 10.1371/journal.pone.0094189

**Published:** 2014-04-09

**Authors:** Tae Nyun Kim, Joo Eun Lee, Eun Ju Lee, Jong Chul Won, Jung Hyun Noh, Kyung Soo Ko, Byoung Doo Rhee, Dong-Jun Kim

**Affiliations:** 1 Department of Internal Medicine, Haeundae Paik Hospital, College of Medicine, Inje University, Busan, Korea; 2 Cardiovascular and Metabolic Disease Center, Inje University, Busan, Korea; 3 Department of Ophthalmology, Haeundae Paik Hospital, College of Medicine, Inje University, Busan, Korea; 4 Department of Internal Medicine, Ilsan-Paik Hospital, College of Medicine, Inje University, Koyang, Korea; 5 Department of Internal Medicine, Sanggye Paik Hospital, College of Medicine, Inje University, Seoul, Korea; Postgraduate Medical Institute & Hull York Medical School, University of Hull, United Kingdom

## Abstract

**Objective:**

We examined the prevalence of and factors associated with lens opacities in a Korean adult population with and without diabetes.

**Research Design and Methods:**

Among the 11,163 adults (≥19 years old) from the fourth Korea National Health and Nutrition Examination Survey in 2008–2009, the data from laboratory tests, nutritional surveys, and slit-lamp examinations of 10,248 persons (4,397 men, 5,851 women) were examined. Cataract was defined as the presence of any nuclear, cortical, subcapsular, or mixed cataract in at least one eye, using the Lens Opacities Classification System III.

**Results:**

The weighted prevalence of cataracts were 23.5% [95% confidence interval (CI), 21.7–25.4] in a Korean adult population (19–39 years old, 1.8% [1.3–2.5], 40–64 years old, 25.2% [22.5–28.1],≥65 years old, 87.8% [85.4–89.9])and 54.7% [50.1–59.2] in a diabetic population(19–39 years old, 11.6% [4.5–26.5], 40–64 years old, 41.1% [35.4–47.0], ≥65 years old, 88.3% [83.5–91.8]). In a logistic regression analysis, age, myopia, and the presence of diabetes were independent risk factors. For young (age 19–39 years) and middle aged (age 40–65 years) adults with diabetes, the OR of having a lens opacity is 5.04 [1.41–17.98] and 1.47 [1.11–1.94], respectively, as those without diabetes, whereas for adults aged 65 and older, there was no difference in the prevalence of cataract.

**Conclusions:**

According to these national survey data, ∼ 24% of Korean adults and ∼ 55% of people with diabetes have cataracts. The presence of diabetes was independently associated with cataracts in young and middle aged adults.

## Introduction

Diabetes is associated with a higher prevalence of cataracts, one of the principal causes of blindness and visual impairment worldwide [1], [2]. Cataracts are characterized by opacification of the translucent media of the lens that progresses rapidly in patients with diabetes. Despite cataracts posing a major public health problem, physicians who manage patients with diabetes may not be familiar with them [3].

In Asia, data on the epidemiological features of cataracts in the diabetic population are limited. Although large-scale population-based studies have found that subjects with diabetes have an approximately five-fold risk of cataracts with cortical and/or posterior subcapsular opacities compared with those without diabetes [4]–[8], these previous studies were conducted mainly in the United States, Australia, Europe, and Africa. Racial differences in the epidemiology of different types of cataracts have been observed [9]–[11]. Additionally, there are significant differences in the characteristics of diabetes between Asian countries and other regions. The incidence and prevalence of type 1 diabetes are lower in Korea than in Western countries [12]. On the other hand, the number of people with type 2 diabetes continues to increase throughout Asia. Individuals in Asia also tend to develop diabetes at younger ages than those in other regions [13].

Cataracts occur at a younger age and progress more rapidly in individuals with diabetes, resulting in higher rates of cataract surgery at a relatively young age [3], [14]. Nevertheless, most population-based surveys of vision and ocular disease have been conducted in middle-aged or older adult populations, with few participants under 40 years old [4]–[8].

The Korean Center for Disease Control and Prevention (CDC) has conducted a series of Korea National Health and Nutrition Examination Surveys (KNHANES) since 1998. In the fourth KNHANES (2007–2009), ophthalmological interviews and examinations were conducted by Epidemiologic Survey Committee of the Korean Ophthalmological Society between 2008 and 2009. In the present study, we examined the prevalence of cataracts in a diabetic population as well as in the entire population in a nationwide representative sample aged 19 years and older from the KNHANES (2008–2009). We also compared the prevalence of cataract subtypes between populations with and without diabetes and investigated the determinants of cataracts.

## Research Design and Methods

### Ethics Statement

This study was approved by the Institutional Review Board of Ilsan Paik Hospital, South Korea (IB 1308–032). After approval of the study proposal, the KNHANES dataset was made available at the request of the investigator. Because the dataset did not include any personal information and the participants’ consent had already been provided for KNHANES, our study was exempted from participant consent by the IRB.

### Study Population and Data Collection

KNHANES is a cross-sectional and nationally representative survey of the health and nutritional status of the civilian, non-institutionalized Korean population. The Korean CDC conducted a KNHANES series in 1998, 2001, 2005, and 2007–2009. In the fourth KNHANES (2007–2009), an annual total of 4,600 households was selected, and ophthalmological examinations were conducted starting in 2008. Participants were selected among candidates using proportional allocation-systemic sampling with multistage stratification based on geographical region, gender, and age group. The participation rate for health examinations (including laboratory tests) in KNHANES in 2008 was 74.3% and that in KNHANES in 2009 was 79.2%. Of these participants, 97% also underwent eye examinations. Among the participants who completed the ophthalmological examination, 10,345 that were>19 years old were included in the analysis.

### Health Examination Survey and Laboratory Tests

Height and weight were obtained using standardized techniques and equipment. Height was measured to the nearest 0.1 cm using a portable standiometer (Seriter, Bismarck, ND). Weight was measured to the nearest 0.1 kg using a Giant–150N calibrated balance-beam scale (Hana, Seoul, Korea). Body mass index (BMI) was calculated by dividing the weight by the height squared (kg/m^2^). Systolic blood pressure (SBP) and diastolic blood pressure (DBP) were measured by standard methods using a sphygmomanometer with the patient in a sitting position. Three measurements were made on all subjects at 5-min intervals; the average of the second and third measurements was used for the analysis. Blood samples were collected in the morning after fasting for at least 8 h. Fasting plasma glucose (FPG), total cholesterol (TC), triglyceride (TG), and high-density lipoprotein cholesterol (HDL-C) levels were measured in a central and certified laboratory, using an Advia 1650 (Siemens, USA). Low-density lipoprotein cholesterol (LDL-C) was estimated indirectly, using the Friedwald formula: LDL-C = TC – HDL-C – (TG/5), for subjects with TG levels <400 mg/dL. Diabetes was defined as fasting plasma glucose ≥126 mg/dL, use of current anti-diabetes medication, or a previous diagnosis of diabetes by a doctor.

### Ocular Examination Protocol

The standardized Lens Opacities Classification System (LOCS) III photographic images were used to assess cataracts [15], [16]. Slit-lamp microscopic examinations (BM-900; Haag-Streit, Koeniz, Switzerland) were performed by board-certified ophthalmologists or ophthalmologists in training. The quality of the survey was verified by the Epidemiological Survey Committee of the Korean Ophthalmological Society [17]. Training for participating ophthalmologists was performed periodically by the National Epidemiological Survey Committee of the Korean Ophthalmological Society. Cataracts were classified as nuclear (LOCS III score ≥4 for nuclear opalescence or nuclear color), cortical (LOCS III score ≥2 for cortical cataracts),posterior subcapsular (PSC, LOCS III score ≥2 for PSC), and mixed cataracts(more than one type per eye) compared with standard photographs [18]. All cataracts were defined as the presence of any one or more type of cataract. History of a previous lens operation (aphakia or pseudophakia) was also recorded and was regarded as presence of a cataract for statistical purposes. Diabetic retinopathy was diagnosed based on fundus photographs, which had been taken using a non-mydriatic fundus camera (TRC-NW6S, Topcon, Tokyo, Japan). If the participants had fasting plasma glucose ≥126 mg/dL, a current anti-diabetes medication, or a previous diagnosis of diabetes by a doctor, seven standard photographs from the Early Treatment for Diabetic Retinopathy Study were obtained from each eye after pharmacological pupil dilatation [19]. Diabetic retinopathy was recorded if the patients had one or more retinal microaneurysms or retinal blot hemorrhages with or without other findings usually seen in patients with diabetic retinopathy. Refractive error was examined using an autorefractor keratometer (KR8800; Topcon, Tokyo, Japan). Spherical equivalents of −0.75 diopters (D) or worse was deemed to be myopia, while +1.0 D or worse was considered hyperopia.

### Statistical Analyses

Data are presented as means or percentages (95% confidence interval [CI]). Participants in the Korean NHANES were not sampled randomly. Specifically, KNHANES was designed with a complex, stratified, multistage probability-sampling model. Thus, each participant does not have the same power in their representation of the whole Korean population. If we try to present a prevalence in the whole Korean population from the dataset, we should consider how much power each participant has for representation (sample weight) of the whole Korean population. After approval of an investigator’s proposal by the Korean Center for Disease Control, the latter provided a survey dataset including information about survey location, strata by age, gender, and other factors, and sample weights for each participant to the investigator. Survey sample weights, calculated by taking the sampling rate, response rate, and age/gender proportions of the reference population (2005 Korean National Census Registry) into consideration, were used in all analyses to produce estimates representative of the non-institutionalized Korean civilian population. Age and age-adjusted comparisons of clinical characteristics according to the presence of cataracts were performed using analysis of covariance (ANCOVA). We used ANCOVA to estimate the prevalence of different types of cataracts by diabetes presence and the prevalence of cataracts in diabetic patients by diabetes duration after adjustment for several variables. To determine which parameter(s) was associated with the presence of cataracts, multiple logistic regression analysis was used. Statistical analyses were performed using the SPSS software (ver. 18.0 for Windows; SPSS, Chicago, IL, USA). Two-tailed analyses were conducted, and *P*<0.05 was deemed to indicate statistical significance.

## Results

Table 1 presents the estimated prevalence of cataracts in persons with diabetes ≥19 years and older and in the whole adult population in Korea. While the weighted prevalence of cataracts was 23.5% [95% CI, 21.7–25.4] in a Korean adult population (19–39 years old, 1.8% [1.3–2.5], 40–64 years old, 25.2% [22.5–28.1], ≥65 years old, 87.8% [85.4–89.9]), the prevalence in the Korean adult diabetic population was 54.7% (19–39 years old, 11.6% [4.5–26.5], 40–64 years old, 41.1% [35.4–47.0], ≥65 years old, 88.3% [83.5–91.8]). Women showed a higher prevalence of cataracts than men in both the whole adult population and diabetes subgroup. However, after adjustment for age, the prevalence of cataracts in women was not different from that in men (24.0±0.7% in women vs. 23.0±0.9% in men, *P = *0.498 in whole population; 56.0±2.8% in women vs. 54.0±2.6 in men, *P = *0.566 in diabetes population).

**Table 1:** Estimated prevalence of lens opacity in theKorean diabetic population and adult population by age and gender.

	Diabetic population in Korea			Korean population		
	Unweighted prevalence	Weighted prevalence	*P*	Unweighted prevalence	Weighted prevalence	*P*
	% (*n* = 986)	% [95% CI] (*n* = 2,732,586)		% (*n* = 10,248)	% [95% CI] (*n* = 34,047,004)	
Total % (*n*)	64.6 (637)	54.7 [50.1–59.2] (1,495,533)		32.5 (3,328)	23.5 [21.7–25.4] (7,993,560)	
Age, years			<0.001^a^			<0.001^a^
19–39	9.4	11.6 [4.5–26.5]		1.8	1.8 [1.3–2.5]	
	(5/53)	(24479/211621)		(60/3415)	(259216/14259805)	
40–64	47.6	41.1 [35.4–47.0]		29.0	25.2 [22.5–28.1]	
	(234/492)	(656754/1598768)		(1351/4659)	(3881157/15399620)	
≥65	90.2	88.3 [83.5–91.8]		88.2	87.8 [85.4–89.9]	
	(492/441)	(814300/922196)		(1917/2174)	(3853186/4387578)	
Gender			0.006^b^			<0.001^b^
Male	61.4	49.7 [43.8–55.6]		31.9	21.7 [19.6–23.8]	
	(291/474)	(731946/1473103)		(1404/4397)	(3678781/16984758)	
Female	67.6	60.6 [54.5–66.4]		32.9	25.3 [23.3–27.3]	
	(346/512)	(763587/1259483)		(1924/5851)	(4314778/17062246)	

a *P* for difference of weighted prevalence among three age group.

b *P* of weighted prevalence between each gender.

Table 2 summarizes age- and gender-adjusted clinical characteristics by the presence of lens opacity. Education level, household income, daily caloric intake, body mass index, and serum total cholesterol levels were lower in persons with cataracts than those without cataracts. In contrast, serum fasting glucose levels were higher in the cataract group than the group without cataracts (98.4±0.9 mg/dL vs. 96.1±0.3 mg/dL, respectively; *P* = 0.030). In addition, the prevalence of myopia, hypertension, diabetes, and diabetic retinopathy were higher in the cataract group.

**Table 2:** Age- and gender-adjusted clinical characteristics by the presence of lens opacity.

	Normal lens (*n* = 6,920)	Cataract (*n* = 3,328)	*P*
Weighted *n*	26,053,444	7,993,560	
Age (years)	38.9±0.3	62.8±0.4	<0.001
Men (%)	51.1±0.6	46.0±1.0	<0.001
Urban living (%)	81.8±2.0	76.4±3.1	0.050
College education	32.1±1.1	22.5±1.2	<0.001
Household income (×1000 KRW/month)	332.5±12.8	274.3±23.8	0.038
Current smoking (%)	26.1±0.6	28.8±1.2	0.062
Heavy alcohol drinking (%)	7.0±0.3	7.5±0.9	0.632
Regular exercise (%)	14.5±0.6	12.8±1.1	0.183
Daily caloric intake (kcal/day)	1935.6±13.1	1860.3±27.6	0.021
Body mass index (kg/m^2^)	23.7±0.1	23.3±0.1	<0.001
Hypertension (%)	20.6±0.6	26.5±1.3	<0.001
Serum total cholesterol (mg/dL)	186.9±0.5	182.9±1.1	0.001
Serum triglyceride (mg/dL)	134.7±1.7	132.3±3.6	0.588
Serum HDL-cholesterol (mg/dL)	52.2±0.2	52.4±0.4	0.624
Fasting plasma glucose (mg/dL)	96.1±0.3	98.4±0.9	0.030
Diabetes (%)	6.7±0.4	12.3±1.0	<0.001
Diabetic retinopathy (%)	0.5±1.2	2.3±3.8	<0.001
Myopia (%)	48.4±0.7	52.5±1.5	0.021

Data, mean ± SEM. Heavy alcohol drinking, ≥ 4 alcoholic drinks/week. Regular exercise, ≥ 5 times/week. Hypertension: systolic blood pressure ≥ 140 mmHg, diastolic blood pressure ≥90 mmHg, or use of current anti-hypertensive medication. Diabetes: fasting plasma glucose ≥ 126 mg/dL, use of current anti-diabetes medication, or a previous diagnosis of diabetes by a doctor.

Table 3 shows the distribution of cataract prevalence (cortical, nuclear, mixed, and any) in the presence and absence of diabetes. Cataract prevalence differences based on the presence of diabetes were significant for the nuclear type and any cataract type individuals in the study population. The any cataract type prevalence was higher in the diabetes group than the non-diabetes group, even after controlling for age, gender, BMI, college education, serum total cholesterol, total daily caloric intake, and the presence of hypertension and myopia. After adjusting for potential variables, the nuclear type was borderline significantly higher in the diabetic group than in the non-diabetic group (15.6±1.9% vs. 12.2±0.7%; *P* = 0.053). Furthermore, more lens operations were observed in the diabetic group than in the non-diabetic group. In the diabetic subgroup analysis, cataract prevalence was highest (66.0±3.9%) in the group with the longest disease duration (>10 years) and lowest (49.0±3.0%) in the group with newly detected diabetes (Table 4).

**Table 3:** Weighted prevalence (%) for different types of lens opacity by the presence of diabetes.

	Total population		Non- diabetes	Diabetes	*P*
Any type	23.5±0.9	Model 1	23.0±0.7	31.0±1.9	<0.001
		Model 2	24.0±0.8	30.0±1.9	0.002
Cortical	5.4±0.5	Model 1	5.3±0.5	5.6±1.1	0.852
		Model 2	5.7±0.5	6.0±1.3	0.906
Nuclear	11.9±0.8	Model 1	11.5±0.7	16.0±1.9	0.011
		Model 2	12.2±0.7	15.6±1.9	0.053
Subcapsular	1.1±0.1	Model 1	1.1±0.1	1.8±0.7	0.331
		Model 2	1.2±0.2	1.4±0.7	0.785
Mixed	3.1±0.3	Model 1	3.1±0.3	3.9±0.9	0.351
		Model 2	3.3±0.3	4.0±1.0	0.509
Past lens operation	4.1±0.3	Model 1	3.8±0.2	7.5±1.1	0.001
		Model 2	4.1±0.3	7.3±1.2	0.014

Data, mean ± SEM.

Model 1: age and gender.

Model 2: model 1+ college education, hypertension, serum cholesterol, BMI, total daily caloric intake, myopia.

**Table 4:** Weighted prevalence (%) of lens opacity in diabetic patients by diabetes duration.

	Newly detected	≤ 5 years	5< and ≤ 10	>10	*P* for trend
Model 1	48.0±3.2	55.0±3.0	55.0±4.0	67.0±4.0	0.002
Model 2	49.0±3.0	56.0±3.1	54.0±4.3	66.0±3.9	0.009

Data, mean ± SEM.

Model 1: age and gender.

Model 2: model 1+ college education, hypertension, serum cholesterol, BMI, total daily caloric intake, myopia.

Table 5 shows the results of multiple logistic regression analyses for risk factors for lens opacity in the Korean adult population, with age, gender, BMI, college education, serum total cholesterol levels, and presence of hypertension, diabetes, and myopia as covariates. For any cataract type, a 10-year increase in age (OR = 4.49, 95% CI = 4.06–4.98), myopia (OR = 1.26, 95% CI = 1.05–1.52), and the presence of diabetes (OR = 1.47, 95% CI = 1.15–1.88) were risk factors, whereas college education (OR = 0.69, 95% CI = 0.56–0.85) was protective. Patients with diabetes had an increased risk for cataract that varied by age (Table 6). For young (age 19–39 years) and middle aged (age 40–65 years) adults with diabetes, the OR of having a lens opacity is 5.04 (95% CI = 1.41–17.98) and 1.47 (95% CI = 1.11–1.94), respectively, compared with those without diabetes, whereas for diabetic subjects aged 65 and older, OR of any cataract is 1.19 (95% CI = 0.79–1.80). When diabetic retinopathy was added as an additional covariate, diabetic retinopathy was significantly associated with the presence of cataracts (OR = 2.51, 95% CI = 1.15–5.45; data not shown).

**Table 5:** Logistic regression analyses for lens opacity in the Korean adult population.

	Odds ratio (95% CI)	*P*
Age (10-year increase)	4.49 (4.06–4.98)	<0.001
Women	1.04 (0.90–1.21)	0.587
College education	0.69 (0.56–0.85)	0.001
Body mass index (1 kg/m^2^ increase)	0.99 (0.96–1.01)	0.248
Hypertension	1.08 (0.92–1.28)	0.355
Serum cholesterol (20 mg/dL increase)	1.02 (0.98–1.06)	0.321
Diabetes	1.47 (1.15–1.88)	0.002
Myopia	1.26 (1.05–1.52)	0.013

Hypertension: systolic blood pressure ≥ 140 mmHg, diastolic blood pressure ≥ 90 mmHg, or use of current anti-hypertensive medication. Diabetes: fasting plasma glucose ≥ 126 mg/dL, use of current anti-diabetes medication, or a previous diagnosis of diabetes by a doctor.

**Table 6:** Odds ratio for lens opacity by the presence of diabetes in different age group.

Age, years	Odds ratio (95% CI)	*P*
19–39	5.04 (1.41–17.98)	0.013
40–64	1.47 (1.11–1.94)	0.008
≥65	1.19 (0.79–1.80)	0.400

Covariates: age, sex, college graduation, body mass index, hypertension, serum cholesterol, myopia.

## Discussion

This study provides epidemiological data on the prevalence of cataracts in a nationwide representative sample 19 years of age and older from the KNHANES (2008–2009). We found that the prevalence of cataracts in the whole Korean adult population was ∼ 23.5% and in the Korean adult population with diabetes ∼ 54.7%. In particular, even though the prevalence of cataracts was similar between the diabetic population 65 years of age and older and individuals without diabetes, cataracts were approximately five-fold more frequent in young (age <40 years) subjects with diabetes than in those without diabetes. Age, college education, and presence of diabetes and myopia were independently associated with the presence of cataracts in the Korean adult population. Our study further showed that even after adjusting for age and gender, there was a significantly higher prevalence of any type and nuclear cataracts and of past cataract surgery in the Korean adult population with diabetes compared with those without diabetes.

Few population-based studies in Korea have reported on cataract prevalence in diabetes. Kim et al. reported that 50.0% of diabetic patients had cataracts at a single Korean diabetes center [20]. Although the prevalence of 54.7% for cataracts in the diabetic population found in our study may appear to be comparable with that reported by Kim et al., the mean age of all patients in their study was 58.07±8.60 years, with few participants below the age of 40 years. Substantial differences in the age-adjusted prevalence of cataracts may exist between hospital-based data and that obtained from large-scale population studies, which are more accurate and can represent entire populations.

Direct comparisons with large-scale population studies in other countries may not be appropriate due to differences in races, the age structure of the population examined, and methodology of cataract assessment. However, a review of literature suggests our prevalence of cataract may be comparable with those reported elsewhere in non-Korean populations. In the United States, the prevalences of self-reported cataracts were 3.8, 11.92, and 38.37% in adults with diabetes of 18–44, 45–64, and ≥65 years of age, respectively, compared with 0.48, 1.58, and 16.56% in non-diabetic adults of the same age groups [2]. Although prevalence of cataract in the present study seems to be higher than among the US population, young and middle aged patients with diabetes might be at greatest risk of cataract in both Korean and US populations. When considering the definition of cataracts using the LOCS III, the overall rate of cataracts in the diabetic population in the present study appeared to be similar to those documented in Asian populations, including Chinese, Singaporeans, and Indians [21]–[23].

Several types of lenticular opacities develop in subjects with diabetes. The distribution of the different types of lens opacities in our whole and diabetic populations should be studied further. In the Korean diabetic population, the age- and gender-adjusted prevalence of nuclear cataracts (16.0%) was higher than that of cortical cataracts (5.6%), with posterior subcapsular cataracts (1.8%) being the least common type. This relative distribution of cataracts in the diabetic population appears to be consistent with that reported in Kinmen, Taiwan, in which nuclear cataracts were most common, followed by cortical cataracts [21], but is dissimilar to an Indian study [23]. In that study, even though the percentage of posterior subcapsular cataracts (1.1%) was comparable with that in the present study, in contrast, the prevalence of cortical cataracts (15.1%) was higher than that of nuclear cataracts (5.0%).

In European and African populations, diabetes has consistently been identified as a risk factor for age-related posterior subcapsular cataracts and cortical, but not likely nuclear, cataracts [4], [5], [7], [24]–[29]. In addition, the Los Angeles Latino Eye Study, a cross-sectional study performed by Richter et al. did not demonstrate an association between the presence of diabetes and nuclear-only lens opacity, whereas diabetes was found to be a significant risk factor for posterior subcapsular cataract-only lens opacity [30]. However, in their 4-year follow-up study, they showed that the presence of diabetes was a significant risk factor associated with development of nuclear-only lens opacity, cortical-only lens opacity, and mixed-type lens opacity. In contrast, the presence of diabetes was not identified as a predictor of posterior subcapsular-only lens opacity [31]. In the present study, the diabetic group had a higher prevalence of nuclear cataracts than did the non-diabetic group even after controlling for potential variables, whereas there was no difference in the prevalence of posterior subcapsular cataracts between the diabetic population and their non-diabetic counterparts.

Although the mechanism for developing cataract in diabetes is poorly understood, diabetes is a well-known risk factor for the development of cataracts [32]. Growing evidence indicates that both the duration of diabetes and the quality of glycemic control are the most important risk factors for diabetic cataract formation [2], [20]. The present study showed that subjects with cataracts had higher fasting blood sugar levels and higher prevalences of diabetes and diabetic retinopathy than did those without cataracts. Additionally, patients with a longer duration of diabetes had a higher prevalence of cataracts than did those with newly-diagnosed diabetes and a shorter duration of diabetes, after adjusting for potential variables. Likewise, hyperglycemia plays a key role in diabetes-associated changes in lens metabolism and cataract formation, whereas other factors known to contribute to diabetic vascular complications, such as hypertension, increased fatty acids, or dyslipidemia, are not important [2]. In multiple logistic regression analysis, in addition to age, we also found that diabetes and myopia, but not hypertension or hypercholesterolemia, were independent risk factors for lens opacities. Myopic eyes are less likely to have diabetic retinopathy but more likely to have nuclear cataracts [30], [33]. Potential mechanisms of myopia leading to nuclear cataracts are largely unknown, but some have hypothesized that axial myopia is a causal factor with a longer vitreous cavity causing decreased diffusion of nutrients to the posterior lens, thus inhibiting oxidative defense mechanisms [30]. Although our data do not provide an explanation for the higher prevalence of nuclear cataracts in this study, both myopia and diabetes may contribute to some degree.

A strength of this study is that we recruited large numbers of participants under 40 years old from the general population. In the present study, among young population, patients with diabetes had five times the risk of cataract compared with subjects without diabetes. The present study has several limitations. Our data were cross-sectional and thus it is not possible to impute causation based on our findings. Additionally, because we studied only a Korean population, caution is needed in generalizing these results to other populations. However, despite a cross-sectional study in a Korean population, accurate epidemiological information may contribute to the proper delivery of healthcare and preventative screenings in individuals with cataracts. This study provides standardized protocols for the examination of ocular diseases and improvements in ocular examination capacity through education and quality control. Finally, the present study was not designed to address the potential mechanisms underlying the association between cataracts and poor glycemic control in patients with diabetes. However, our findings suggest that glycemic control may play an important role in explaining the increased risk of cataracts with regard to diabetes.

In conclusion, this is the first reported study investigating the prevalence of diabetic cataracts in Korea, based on nationally representative data. The prevalence of cataracts in the whole Korean adult population is ∼ 23.5% and that in the Korean adult population with diabetes is ∼ 54.7%. Additionally, the present results provide some evidence that patients with diabetes are significantly disadvantaged with respect to lens transparency and may be at greatest risk of developing cataracts in young adult patients with diabetes.
